# Appendiceal diverticulitis and inflammatory bowel disease

**DOI:** 10.1093/jscr/rjac586

**Published:** 2023-01-18

**Authors:** Martha Lok-Yung Hui, Justin Ho-Yin Ng, Olivia Cummings, Nelson Chen

**Affiliations:** General Medicine, Bendigo Health, Bendigo, Victoria 3564, Australia; General Surgery, Echuca Regional Health, Echuca, Victoria 3564, Australia; Surgical Science, University of Edinburgh, Edinburgh EH89YL, UK; General Medicine, Bendigo Health, Bendigo, Victoria 3564, Australia; General Surgery, Echuca Regional Health, Echuca, Victoria 3564, Australia; General Surgery, Echuca Regional Health, Echuca, Victoria 3564, Australia; General Surgery, Austin Health, Heidelberg, Victoria 3564, Australia

## Abstract

Appendiceal diverticulitis is known as a rare pathology and its etiology remains largely unknown. We describe a case of a 41-year-old woman with a past history of inflammatory bowel disease (IBD) who was admitted to the Emergency Department at a rural hospital in Australia with right iliac fossa pain (RIF) and later was found to have acute appendiceal diverticulitis on histopathologic studies. Thus far, no literature has described IBD as one of the possible contributing factors of appendiceal diverticulitis. This paper aims to shed light on the possible causative relation between appendiceal diverticulitis and IBD.

## INTRODUCTION

Appendiceal diverticulitis is a rare disease that was first described by Kelynac in 1893 [1]. It often mimics acute appendicitis because of their similar clinical features [1], and diagnosis is often overlooked or made only after surgery with pathological examination, which causes delay in treatment and thus increases the risk of serious complications. Appendiceal diverticulitis carries much higher risks of perforation and therefore more suspicion and attention should be paid to allow immediate interventions. In this paper, we present the case of a 41-year-old woman with appendiceal diverticulitis on a background history of inflammatory bowel disease (IBD). We aim to explore the role of IBD in the etiology of appendiceal diverticulitis.

## CASE REPORT

A 41-year-old female presented to the Emergency Department of a rural hospital in Australia with a 1-day history of severe right lower quadrant pain associated with fevers, nausea and vomiting. She also has had ongoing diarrhea and nausea for 3 months, which were associated with a weight loss of 4 kg. Colonoscopy was performed 1 month ago, identifying an unremarkable appendiceal orifice, a few small sigmoid diverticulae and three sessile polyps that were removed with cold-snare polypectomy. Her medical history was significant including IBD, hemorrhoids, medullary sponge kidney and endometriosis. She was not on any regular medications and had a 10 pack-year smoking history.

**Figure 1:**
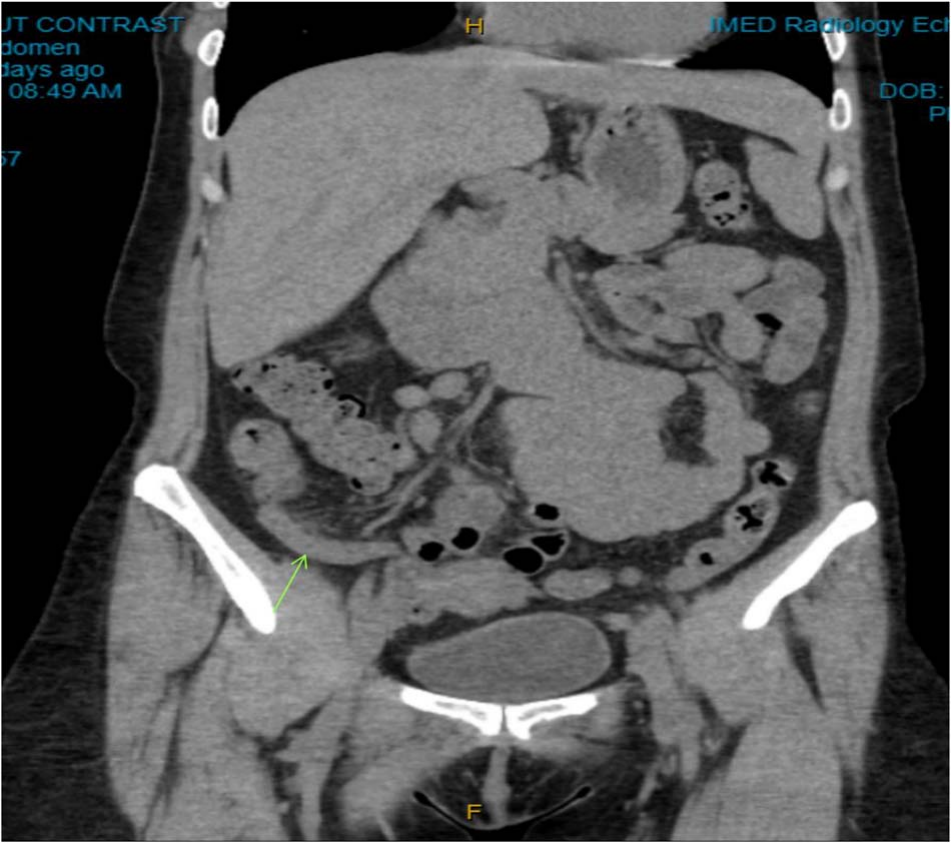
Coronal CT image. Dilated appendix with mild surrounding fat stranding.

**Figure 2:**
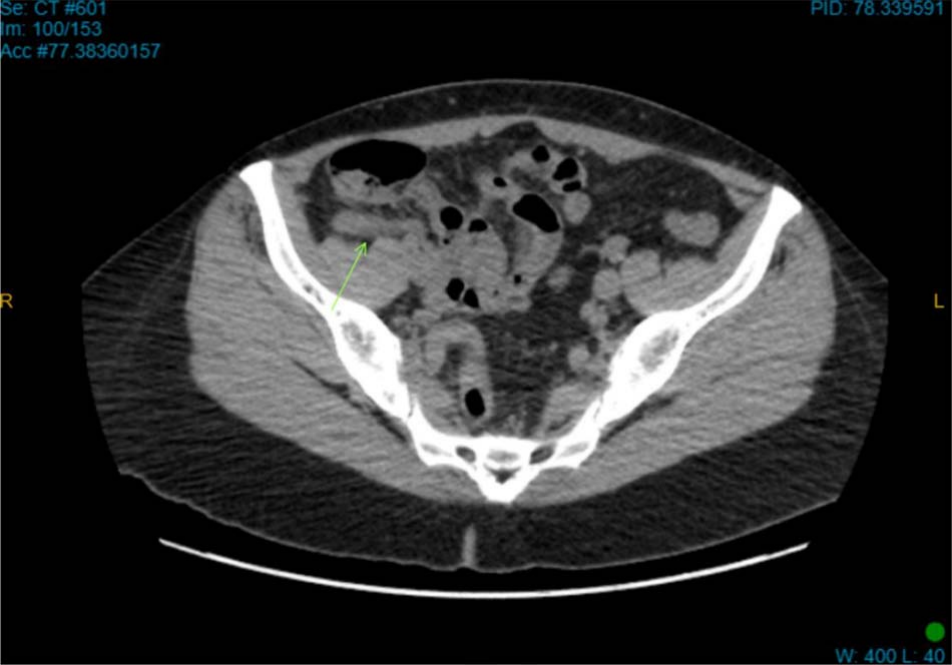
Axial CT image. Retro-cecal inflamed appendix.

**Figure 3:**
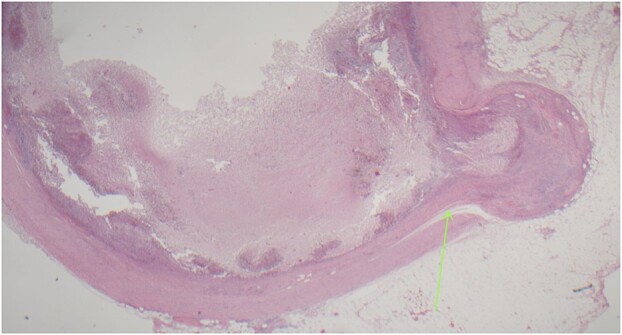
Acutely inflamed appendiceal diverticulum with transmural mixed inflammation extending out into subserosa and serosa.

On examination, the patient was alert, but was grimacing in pain. Her abdomen was soft but extremely tender on the right iliac fossa (RIF) region with positive Rovsing’s sign, and rebound tenderness was noted. Vitals were stable. The white cell count was 16.8 × 109/L with left neutrophil shift. The CRP was 102 mg/L. Other laboratory investigations were unremarkable. A computed tomography (CT) scan of the abdomen showed a thickened appendix with adjacent fat stranding which was consistent with acute appendicitis (Figs 1 and 2). Intravenous Augmentin was given pre-operatively and laparoscopic appendectomy was performed on the same day. The patient recovered uneventfully and was discharged the next day. The histological reports revealed appendiceal diverticulitis with transmural mixed inflammation extending out into subserosa and serosa (Fig. 3).

## DISCUSSION

Appendiceal diverticulitis, first described in medical literature by Kelynack in 1893 [1], is a rare pathology with an incidence rate of 0.004–2.1% [2] that is often mistaken as acute appendicitis. Although their definitive treatment is the same, appendiceal diverticulitis carries a much higher risk of perforation and development of neoplasms than acute appendicitis [3]. The incidence of neoplasia in appendiceal diverticulitis is 48% in which most of them are neuroendocrine tumors [4].

Appendiceal diverticulitis is classified into congenital and acquired types [5]. Acquired type involves herniation of mucosa and submucosa protruding through the defective muscularis, although, in congenital, all four layers of the appendiceal wall are affected [3]. Congenital type is extremely rare with about only 50 cases ever reported in history globally [6]. The majority of cases are of the acquired type. Risk factors for acquired appendiceal diverticulitis include male sex, age greater than 30 years, chronic appendicitis, Hirschprung’s disease and cystic fibrosis [7, 8].The exact pathogenesis of acquired appendiceal diverticulitis is not fully understood, but it is thought to be caused by the increased pressure from an obstruction at the orifice secondary to a appendicolith or a mass, resulting in mucosal herniations [9, 10]. The inflammatory reaction leads to atrophy of the lymphoid tissue, which further weakens the appendiceal wall and subsequently exacerbates the herniation [11].

This case is deemed unusual for the early presentation of alternating bowel habits in appendiceal diverticulitis and there seems to be rarely found in the literature on the subject of chronic symptoms. Studies have shown that the mean duration of symptoms in appendiceal diverticulitis was approximately 3.6 days pre-operatively and diarrhea was only found in 17.3% of subjects [11]. The early chronic diarrhea in this patient is exceptionally uncommon and may be the manifestation of IBD instead, particularly as one of the classic symptoms of IBD [12]. With the background history of IBD, appendiceal diverticulitis may be the result of the pathophysiological event arising from IBD if we look into its physiology and pathogenesis.

Both ulcerative colitis and Crohn’s disease, the two major forms of IBD, are typified by a certain degree of damage to the intestinal mucosa. Physiologically, excessive inflammatory mediators in IBD lead to damage to the architecture of the mucosal wall and result in loss of function of the luminal membrane [12]. Consequently, the epithelial barrier loses its integrity and becomes unable to regulate ion absorption resulting in water retention in the lumen and diarrhea [12, 13]. The pathogenesis of IBD is multifactorial and involves complex interactions between genetic disposition, environmental components, gut flora and immune modulation [14]. It is indicated that the dysfunctions of immune pathways and accumulation of neutrophils in mucosa contribute to the intestinal inflammatory response and damage of lining in IBD [14]. Based on the above, the authors hypothesise that the persistent presence of inflammatory mediators exacerbates the swelling of the appendiceal wall as well as the orifice, which further aggravates the obstruction contributing to appendiceal diverticulitis.

Appendiceal diverticulitis should be taken into consideration as one of the differential diagnoses especially in patients with a change in bowel habits along with RIF tenderness. As appendiceal diverticulitis has a much higher risk of perforation and development of neoplasms than acute appendicitis, early recognition of signs and symptoms is warranted to prevent serious complications and should be considered in older age groups and patients with IBD presenting with symptoms of acute appendicitis. This case report highlights the possibility of IBD being one of the causative factors of appendiceal diverticulitis. The main purpose of the case report is to highlight the potential association between IBD and the pathogenesis of appendiceal diverticulitis. Future research is needed to gain further insights into the relation between the two diseases.
